# Impact of muscle mass on the prognosis of liver transplantation for infants with biliary atresia

**DOI:** 10.3389/fped.2022.1093880

**Published:** 2023-01-16

**Authors:** María D. Lledín, Manuel Parrón-Pajares, Ana Morais, Francisco Hernández-Oliveros, Jose I. Botella-Carretero, Loreto Hierro

**Affiliations:** ^1^ Department of Pediatric Hepatology & Liver Transplant, Hospital Universitario La Paz & IdiPAZ, Madrid, Spain; ^2^ Department of Pediatric Radiology, Hospital Universitario La Paz, Madrid, Spain; ^3^ Department of Pediatric Nutrition, Hospital Universitario La Paz, Madrid, Spain; ^4^ Department of Pediatric Surgery, Hospital Universitario La Paz, Madrid, Spain; ^5^ Department of Endocrinology & Nutrition, Hospital Universitario Ramón y Cajal & IRyCIS, Madrid, Spain; ^6^ European Reference Network on Hepatological Diseases (ERN RARE-LIVER), Hospital Infantil La Paz, Madrid, Spain

**Keywords:** liver transplant, infants, biliary atresia, muscle mass, sarcopenia, pediatrics

## Abstract

**Background:**

Sarcopenia in adult cirrhotic patients is associated with increased morbidity and mortality whereas in children it is still being studied. Anthropometric variables in cirrhotic children are not reliable for assessing muscle mass as they may be altered by ascites, edema, and organomegaly. Measuring the area of the psoas showed good correlation with muscle mass in adults. We aimed to study in cirrhotic infants undergoing liver transplantation the association of the psoas area with liver transplant prognosis as well as with several analytical and anthropometric parameters used to evaluate nutritional status.

**Methods:**

Retrospective cohort of 29 infants with cirrhosis due to biliary atresia who underwent abdominal CT scan as a pre-transplant study. We measured the psoas muscle index (PMI) at L4-L5 since it best correlates with muscle mass in pediatric patients. As there are no validated cut-off points to define sarcopenia in children under one year of age, PMI was recorded as a continuous variable and correlated with different prognostic, clinical, and analytical variables. The SPSS 17.0 package was used for statistical analysis and a *P* < 0.05 was considered significant.

**Results:**

29 infants (10 boys, 19 girls) were studied. 62% were Caucasian and the rest were South American. The mean age at CT scan was 8.5 months (range 3–15 months). There was a negative correlation between PMI and days of admission prior to liver transplant, previous infections, and bone fractures. Among the analytical parameters, cholinesterase, albumin, and prealbumin correlated positively with PMI (*P* < 0.05). No relationship was observed with anthropometric parameters: weight, height, BMI, brachial perimeter, or bioimpedance. During surgery, patients with lower PMI had a greater need for plasma transfusion, and in the immediate postoperative period, there was a longer stay in intensive care, more days of mechanical ventilation, and more days of hospital admission (*P* < 0.05). On the contrary, no relationship was found with other complications: bleeding, re-interventions, biliary leaks, rejection, thrombosis, re-transplantation, or infections.

**Conclusions:**

The decrease in muscle mass is associated with increased morbidity in infants with biliary atresia undergoing liver transplantation. Muscle mass in these patients cannot be adequately assessed with anthropometric measurements commonly used in the clinic.

## Introduction

Biliary atresia (BA) is a progressive cholangiopathy of the intrahepatic and extrahepatic bile ducts, causing neonatal cholestasis with a rapid progression to cirrhosis. Untreated patients die by age of 2 years. The first therapeutic option is a surgical intervention with Kasai portoenterostomy (KPE) for restoring bile flow (1) which provides successful drainage in 30% to 70% of patients (2). Patients without surgery, with failed KPE, or with complications as recurrent cholangitis, malnutrition, portal hypertension with ascites, or variceal bleeding are candidates for liver transplant (LT) (3). Children with age <1 year with BA on the waiting list for LT are a group with a high risk of mortality (4, 5). Malnutrition, among others, is one of the major predictors of poor outcomes both at the pre- and post-LT stages, especially because of muscle mass loss (6, 7).

Sarcopenia was initially defined as a progressive and generalized loss of skeletal muscle mass, strength, and function with risk of adverse outcomes (8). In cirrhotic adult patients, sarcopenia has been studied as a prognostic marker for adverse overall outcomes and was associated with increased mortality and poor liver transplant outcomes (9–11). In the last years, there has been a growing interest of sarcopenia research in the pediatric population. In children, sarcopenia has been associated with poorer growth parameters, increased risk of death before LT and longer intensive care unit (ICU) stay post-LT (12).

Multiple factors contribute to malnutrition and sarcopenia in cholestatic pediatric patients: (a) Decreased energy intake secondary to anorexia, nausea, gastroesophageal reflux disease, vomiting and early satiety; (b) Decreased absorption of fat and fat-soluble vitamins that is aggravated by portal hypertension-induced enteropathy (7); (c) Altered nutrients metabolism with a decrease in protein synthesis and its use, with abnormal plasma amino acid profiles with low levels of branched-chain amino acids; (d) A hypermetabolic state, with higher energy needs above 40% from the basal ones (13); (e) Growth hormone (GH) resistance, with elevated GH levels and low-circulating insulin-like growth factor 1 (IGF-1) and insulin-like growth factor binding proteins (14), which may play a role in the development of sarcopenia in children with BA (15); (f) Physical deconditioning and systemic inflammation that may worsen muscle loss (16, 17); (g) Alterations in microbiome in BA children that may contribute to malnutrition (18).

Therefore nutritional assessment is mandatory in the management of these patients with a close monitoring of their trends over time. Anthropometric measurements are employed in daily care and changes in weight, weight-for-length, and body mass index (BMI) must be interpreted cautiously due to organomegaly, fluctuating ascites, edema, and fluid retention. Other measurements commonly used in children with chronic liver disease are mid-upper arm circumference (MUAC) that reflects lean and adipose mass, and triceps skin fold that reflects adipose tissue (13). Although both have been considered as sensitive markers of malnutrition, their measurement requires trained personnel and should be performed serially to evaluate the adequacy and impact of nutritional interventions (7). Further, in pediatric patients with BA <2 years, computed tomography-based body metrics showed a poor to fair correlation with MUAC (19).

More complex and accurate methods to evaluate body composition are bio-electric impedance analysis (BIA), dual-energy x-ray absorptiometry (DEXA), computed tomography (CT) and magnetic resonance imaging (MRI). BIA is modified by the hydration status, and some differences in intracellular and transcellular penetrations between genders and individuals reduce the consistency and accuracy of this technique (20, 21). DEXA in children <2 years may need sedation, and a lack of normative pediatric data may limit the recognition of sarcopenia (22). Therefore CT has been considered the gold standard for assessing skeletal muscle mass in pediatric population (23).

Owing to the relevance of nutritional status and more specifically the muscle mass in the prognosis of liver transplantation, we aimed to study, in cirrhotic infants undergoing liver transplantation, the association of the psoas muscle area with liver transplant prognosis as well as with several analytical and anthropometric parameters commonly used to evaluate nutritional status.

## Materials and methods

### Study population

We performed a retrospective cohort single-center study at the Hospital Universitario La Paz in Madrid, Spain. Twenty-nine patients younger than 18 months with a diagnosis of BA who underwent LT from January 2015 to December 2019 were studied. Children were excluded if their abdominal CT imaging was not available within 3 months before LT. The study was approved by the Ethics Committee of our Center (Ethics number PI4682, grant PI20/1496) and informed consent was obtained from every participant´s legal guardian/next of kin.

### Data collection

All medical charts were reviewed and the following data were recorded: age, sex, race, restoration of bile flow (defined as bilirubin less than 2 mg/dl, 3 months after KPE). Among pre-LT variables we recorded the following: hospital stay before LT (days), infections pre- LT (cholangitis, peritonitis, and sepsis), ascites, digestive bleeding, bone fractures and pediatric-end stage liver disease score (PELD).

We selected the closest blood test to the CT scan for hematological and biochemical variables, and also the closest nutritional evaluation for which we recorded the following: weight (kilograms), height (meters), MUAC, triceps skin fold, and subscapular fold (the latter three expressed as *Z*-score calculated from pediatric reference charts). BIA was also performed (RJL systems, Clinton Township, MI 48035 US) and phase angle recorded.

Surgical-associated variables included in the data set were: type of graft (deceased or living donor), type of bile duct anatomosis (single or double), risky vascular anastomosis (used of vascular grafting or Tanaka variant for portal reconstruction), any surgical complication during the intervention, use of Gore-Tex patch, time of cold ischemia and anhepatic phase, and volume of transfusions (red blood cells and fresh frozen plasma).

Variables in the post-operative period were: days in ICU, days of mechanical ventilation and noninvasive ventilation, biliary pathology (bilioma, biliary fistula), bleeding, re-operation, rejection episodes, arterial thrombosis, re-transplantation, and post-operative infections. We also recorded the number of re-hospitalizations in the first year post-LT.

### Measurement of muscle mass by CT scan

Abdominal CT images were reviewed by one expert in pediatric radiology (M.P.). From axial CT images, L4–5 level was identified following cross-referencing on sagittal and coronal plane reconstruction. On axial cross-sectional CT images, cross-sectional area of the psoas muscle was measured using a dedicated free-hand drawing tool at 2 separate intervertebral lumbar disks (L4–5) level by PACS software (IMPAX Volume Viewing 4.0. Agfa HealthCare Clinical applications, Mortsel, Belgium).

**Figure 1:**
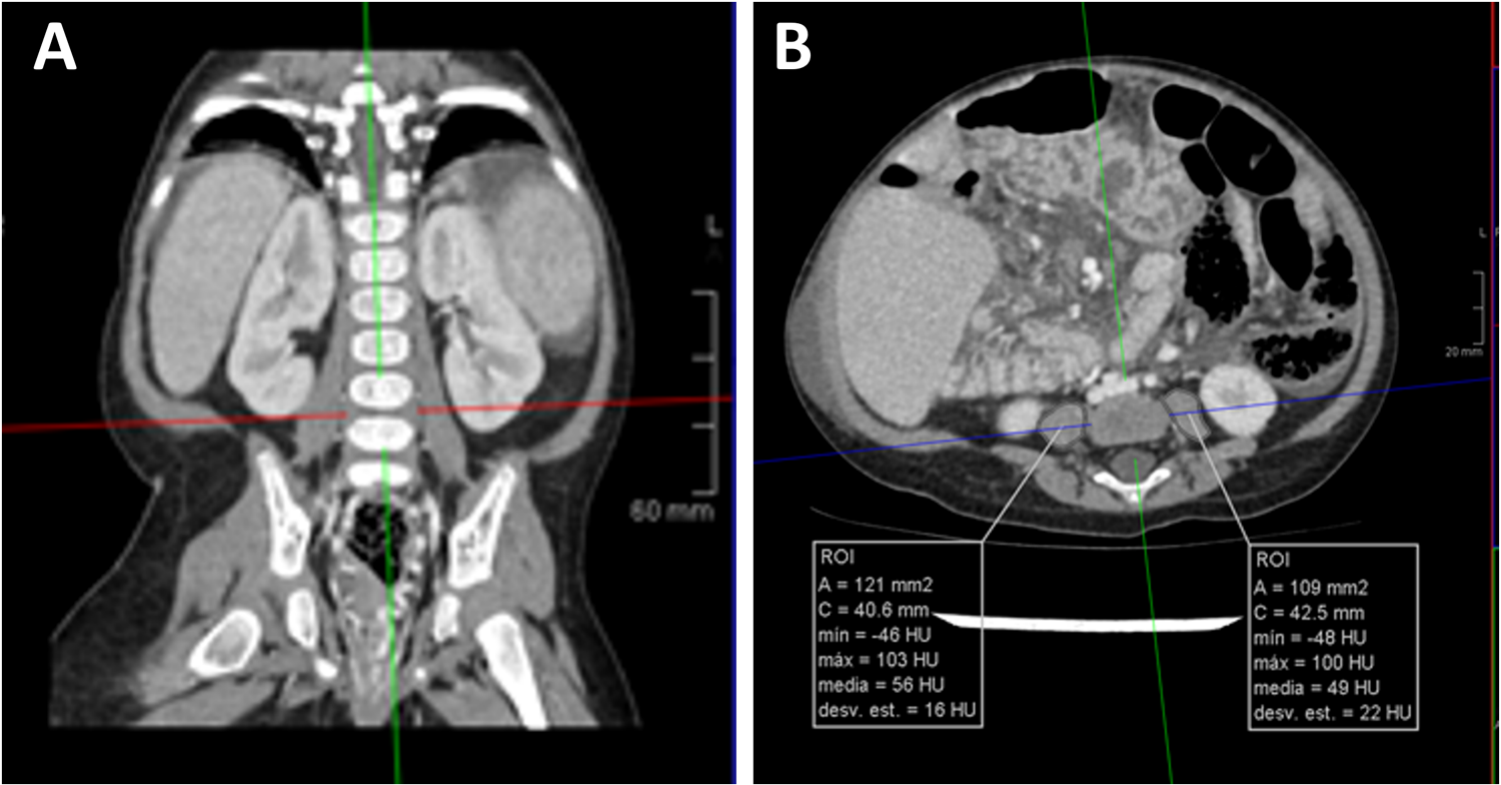
Measurement of psoas muscle mass in CT scan. Panel (**A**). From axial CT images, L4-5 level was identified following cross-referencing on sagittal and coronal plane reconstruction (red line in left image). Panel (**B**). On axial cross-sectional CT images, cross-sectional area of the psoas muscle (white lines in right image) was measured using a dedicated free-hand drawing tool.

Total psoas surface muscle area (tPMA), was expressed as the sum of the right and left PMA in square millimeters (mm^2^) at the same level (24, 25). We performed one measurement at L4–5 level, it has been shown to be the most clinical relevant and useful measure in children (25) (Figure 1). It was divided by height squared and expressed as psoas muscle index (PMI, mm^2^/m^2^). As there are no validated cut-off points for infants as to accurately define sarcopenia, we used PMI as a continuous variable in this study (25).

### Statistical analyses

A priori sample size analysis was performed with the online tool GRANMO 7.12 (https://www.imim.es/ofertadeserveis/software-public/granmo/index.html). A total sample size of 30 subjects was required to detect a correlation coefficient of 0.5, for a bilateral contrast, and with no losses on follow-up, with 1 − *β* = 0.80 and *α* = 0.05.

Results are expressed as means ± SD unless otherwise stated. The Kolmogorov-Smirnov statistic was applied to continuous variables. Logarithmic or square root transformations were applied as needed to ensure a normal distribution of the variables. Comparisons between the different groups at baseline were performed by independent t test for continuous variables or the Mann-Whitney U test for non-normal distributed variables, and by *χ*2 test or Fisher's exact test for discontinuous variables. Bivariate correlation analyzed the association between two continuous variables by Pearson or Spearman's tests. Analyses were performed using SPSS 17 (SPSS Inc, Chicago, Illinois). *P* < 0.05 was considered statistically significant.

## Results

Twenty-nine children (10 boys and 19 girls) were studied. 62% were Caucasian and the rest were South American. All infants were full-term newborns. The mean age at CT scan was 8.5 months (range 3–15 months). All patients underwent LT. The survival of the included children was 96.6% at the end of study (one child died as a result of sepsis at late postoperative period).

**Figure 2:**
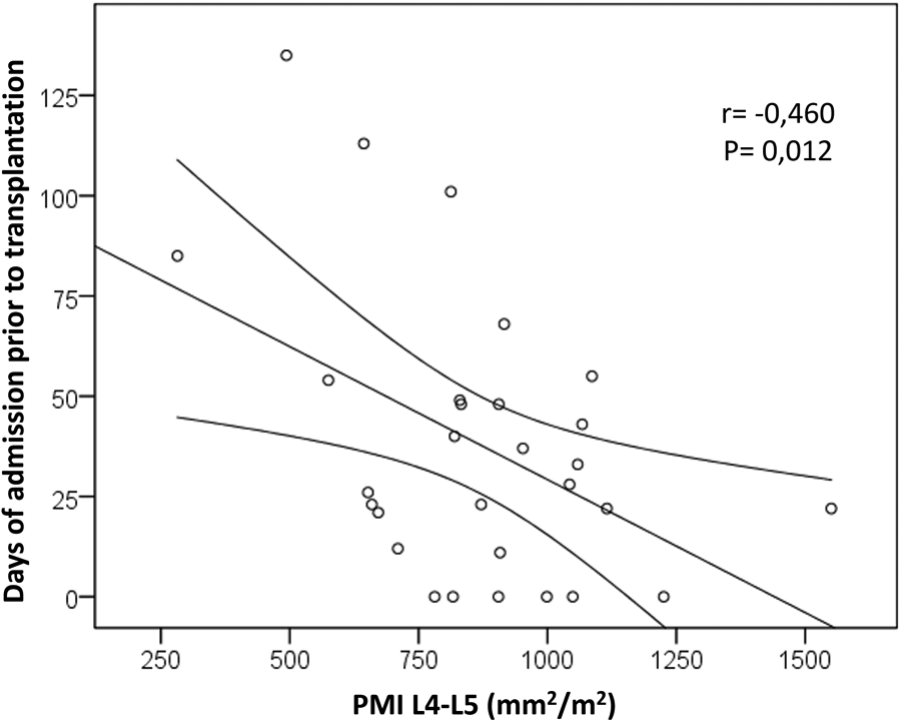
Correlation of psoas muscle mass with pre-transplantation variables. A bivariate negative correlation was found between psoas muscle index (PMI) measured at L4-5 level and the days of admission prior to liver transplantation.

Regarding pre-LT variables, there was a negative association between muscle mass expressed as PMI and preoperative infections (cholangitis, peritonitis and sepsis) and also with the occurrence of bone fractures (Table 1). There was also a negative correlation between PMI and days of admission prior to LT (Figure 2). Among the analytical parameters, cholinesterase, albumin and prealbumin correlated positively with PMI (Table 2). On the other hand, there was no correlation between PMI and several anthropometric measures used in daily clinical practice or with the phase angle measured by BIA (Table 3).

**Table 1:** Comparison of PMI regarding pre-liver transplantation variables.

	Yes	No	*t*	*P*
Restoration of bile flow	*n* = 6 828 ± 182	*n* = 18 895 ± 279	0.54	0.59
Infections (cholangitis, peritonitis, sepsis)	*n* = 20 805 ± 213	*n* = 9 1014 ± 255	2.30	0.029
Ascites	*n* = 17 812 ± 220	*n* = 12 952 ± 260	1.56	0.129
Digestive bleeding	*n* = 2 653.12 ± 225	*n* = 27 886.20 ± 241	1.55*	0.121*
Bone fractures	*n* = 3 450.3 ± 151	*n* = 26 918.57 ± 202	2.78*	0.005*

Data are shown as mean ± SD. PMI, psoas muscle index.

* Calculated using the Mann–Whitney test.

**Table 2:** Correlation of PMI with biochemical parameters before liver transplantation.

	Mean	SD	*r*	*P*
Leukocytes (*n*/mm^3^)	10,986	6,226	−0.326	0.085
Platelets (*n*/mm^3^)	181,070	71,602	0.126	0.518
INR	1.28	0.22	−0.065	0.737
Fibrinogen (mg/dl)	285.24	111.26	0.272	0.154
ALT (IU/L)	188	145	0.167	0.386
GGT (IU/L)	707	554	0.289	0.136
Direct bilirubin (mg/dl)	10.37	5.45	−0.336	0.087
Alkaline phosphatase (IU/L)	858.4	349.3	0.093	0.329
Creatinine (mg/dl)	0.20	0.04	0.008	0.967
Total proteins (g/dl)	5.76	0.64	−0.080	0.687
Cholinesterase (IU/L)	4,023	1,691	0.539	0.030
Albumin (g/dl)	3.3	0.4	0.390	0.036
Pre-albumin (mg/dl)	9.5	3.2	0.478	0.012
Cholesterol (mg/dl)	292.7	210.6	0.182	0.383
Vitamin E/Cholesterol	2.4	1.8	−0.031	0.877
25-OH-vitamin D (µg/dl)	31.42	27.17	−0.024	0.078

PMI, psoas muscle index; INR, international normalized ratio; ALT, alanine amino-transpherase; GGT, gamma glutamyl transpeptidase.

**Table 3:** Correlation of PMI with anthropometric measures and phase angle by BIA.

	Mean	SD	*r*	*P*
Weight (*Z*-score)	−1.41	1.12	0.282	0.138
Height (*Z*-score)	−1.37	1.18	0.376	0.450
Cephalic perimeter (*Z*-score)	−1.46	1.21	0.235	0.281
Weight/Height (*Z*-score)	−0.69	1.15	0.001	0.095
BMI (*Z*-score)	−0.83	1.14	0.250	0.899
MUAC (*Z*-score)	−1.37	0.97	0.322	0.144
Triceps skinfold (*Z*-score)	−0.98	2.17	−0.298	0.203
Subescapular fold (*Z*-score)	−0.72	1.08	−0.145	0.519
Phase Angle	3.15	0.73	0.162	0.507

PMI, psoas muscle index; BIA, bio-electric impedance analysis; BMI, body mass index; MUAC, mid upper arm circumference.

During surgery, patients with lower PMI had a greater need for plasma transfusion, as shown by a negative correlation, whereas no correlation was found with PELD score, time of cold ischemia, time of anhepatic phase, total surgical time or the need for red blood cells transfusion (Table 4). There were no differences with the type of graft (deceased or living donor, *P* = 0.114), type of bile duct (single or double, *P* = 0.254), risky vascular anastomosis (*P* = 0.767), or surgical complications (*P* = 0.620). However, those children who needed a Gore-Tex patch showed lower PMI (Yes: 757.6 ± 234.6, No: 974.89 ± 208.30 mm^2^/m^2^ respectively, *t* = 2.638, *P* = 0.014).

**Table 4:** Correlation of PMI with surgical data.

	Mean	SD	*r*	*P*
PELD (score)	15.10	8.57	−0.272	0.153
Time of cold ischemia (min)	366.11	82.51	−0.245	0.219
Time of anhepatic phase (min)	65.44	31.70	−0.117	0.576
Total time (min)	564.55	111.18	−0.219	0.328
Transfusion of red blood cells (ml/Kg)	103.68	69.52	−0.260	0.181
Transfusion of fresh frozen plasma (ml/Kg)	197.21	91.01	−0.465	**0**.**013**

PMI, psoas muscle index; PELD, pediatric-end stage liver disease score.

**Figure 3:**
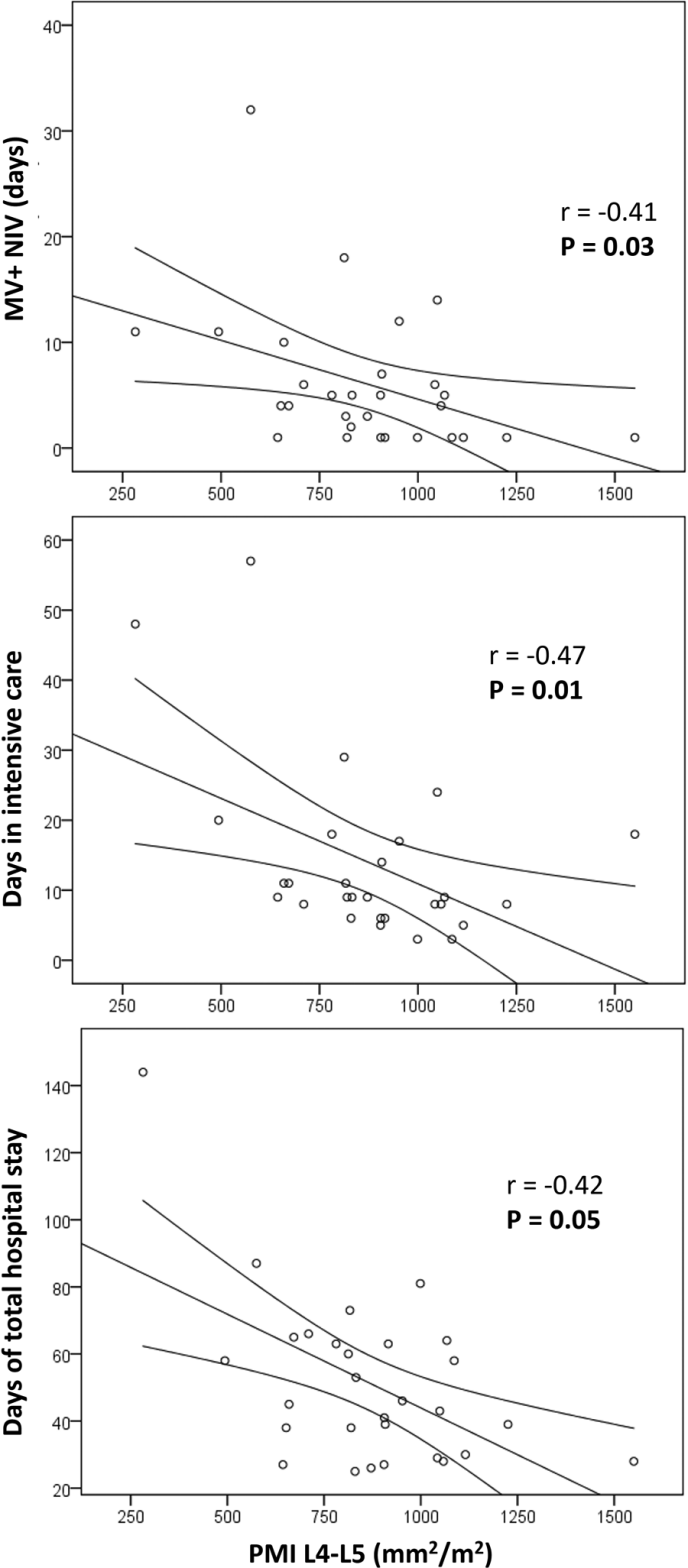
Correlation of psoas muscle mass with post-transplantation variables. There was a negative correlation between psoas muscle index (PMI) and the days of ventilation, both mechanical ventilation and non-invasive ventilation. There was also a negative correlation between PMI and days in ICU and total hospital stay.

Focusing on post-LT complications, we found a negative correlation between PMI and the days of ventilation, both mechanical ventilation and non-invasive ventilation (Figure 3). Conversely, no association was found with other immediate post-LT complications: biliary pathology (*P* = 0.45), bleeding (*P* = 0.72), re-operation (*P* = 0.15), rejection (*P* = 0.34), thrombosis (*P* = 0.57), re-transplantation (*P* = 0.86) or postoperative infections (*P* = 0.25). There was also a negative correlation between PMI and days in ICU (*P* = 0.01) and total hospital stay (*P* = 0.05) (Figure 3), but no relationship with admissions in the first year post-LT. Conversely other nutritional variables such as weight/height, cephalic perimeter, BMI, MUAC, triceps skinfold, subscapular fold and phase angle showed no correlation with days in ventilation, days in ICU, or total hospital stay (*P* > 0.05 for all bivariate correlations).

## Discussion

In this study, we have shown that the decrease in muscle mass in infants with BA submitted to LT was associated with increased morbidity, specifically with longer ICU and total hospital stays and prolonged days of mechanical ventilation. A greater need for plasma transfusion and Gore-Tex patch was also significant, whereas no association was found with other immediate post-LT complications. Interestingly, we found no association of muscle mass with several anthropometric measures used in daily clinical practice or with the phase angle measured by BIA.

The first handicap in our study was trying to establish a cut-off point to define sarcopenia in children under 1 year of age. Previous published studies were based on a control group in this population (13, 26), and recently Lurz et al. published curves of tPMA for a Canadian population of 779 children, but did not include children under 1 year of age (25). Metzger et al. published curves of tPMA in a group of 782 patients in the US and also included a small set of children under 1 year of age, but showing only p25-p50 values and no clear cut-off points (27). As we do not currently have enough abdominal CT images of healthy children under one year of age to include a control group for our population, we decided to study psoas muscle mass as a continuous variable in our statistical analyses. Further, we used PMI to standardize the measurement and avoid differences that might exist in terms of sex, race and body constitution. Although tPMA did not show a significant difference in boys and girls less than 8 years of age (25, 28), PMI represents a metric that is more consistent across ages, especially in infants (27).

Previous studies have used both tPMA and PMI when measuring muscle mass in children submitted to LT. Boster JM et al. (29) found higher mortality rates in those children candidates to LT who had sarcopenia. They performed a cross sectional study in 57 patients with abdominal imaging within 12 months prior to LT, aged from 0 to 18 years, without liver tumor or metabolic liver disease and without fibrosis. They compared them with a control group using tPMA. Their results showed an increased risk of death (either while on the waiting list or following LT) for those with lower muscle mass, but found no correlation of tPMA with ventilation time, hospital length of stay, other peri-transplant complications or with the PELD score (29).

Woolfson JP et al. (12) studied 25 pediatric patients younger than 16 years of age who underwent first isolated LT, but children younger than 1 year were excluded. The grouping of patients with sarcopenia was based on the curves published by Lurz et al. (25). The 10 patients included in the sarcopenia group had a significantly longer duration of ICU stay after LT. They found no differences in vascular or biliary complications, time to discharge, rejection, graft loss or survival at 1 year follow-up (12). A similar report was published by Verhagen MV et al. (30) studying different measurements in CT-imaging (PMI, skeletal muscle index and subcutaneous fat area index) in patients younger than 18 years who underwent a primary LT (*n* = 101). Conversely, these authors reported the results in children <1 year (*n* = 59) with cirrhotic liver disease, and found that skeletal muscle index correlated with longer duration of hospital and ICU stay (30), similar to our results.

Some studies were specifically focused on infants with LT: Takeda et al. (26) studied the influence of sarcopenia on post-LT outcomes in a group of 89 infants with BA. Sarcopenia was defined as tPMA less than −2 SD below the mean of a healthy control group. The 21 patients with sarcopenia showed longer operation time and higher blood loss during surgery, a higher incidence of portal stenosis and post-LT infections. Conversely the total length of hospital stay was not affected (26). Jitwongwai el al (24). showed an association between PMI and waiting list mortality and also with post-LT outcomes. They included 105 children (84% with BA) with a mean age of 12 months (range 10–24 months). CT was performed within 12 months before LT or death on waiting list (mortality rate of 29%). Lower PMI was associated with higher reoperation rate and longer hospital stay following transplantation, but not with waiting list mortality. In this study variceal bleeding, septicemia and bone fractures were significantly associated with low muscle mass after multivariate analyses. Conversely PMI did not correlated with PELD score, *Z*-score of weight for height or BMI (24).

Our study showed similar results to the ones already discussed, with some clear advantages: First we studied a homogeneous group of patients, all of them with a diagnosis of BA and younger than 15 months at LT. Second, the interval from CT scan to LT was within 3 months, and in all of them we also performed a nutritional assessment with many analytical, anthropometric and BIA measurements. Third, we have only analyzed the patients who received a LT and therefore we did not take into account the mortality on the waiting list for LT. This make the results more homogeneous but may underestimate the importance and impact of muscle wasting in all children with BA whether or not they are submitted to LT.

We confirmed that the analytical values that better correlate with muscle mass were serum albumin, prealbumin and cholinesterase, whose values in most cholestatic patients with malnutrition may be altered due to the altered protein synthesis and metabolism by the liver. It is necessary to highlight and emphasize that the anthropometric measures commonly used in clinical practice and different severity scores such as the PELD did not detect children with loss of muscle mass and, as we also demonstrated, these patients were more vulnerable and showed a higher risk of pre- and post-LT complications. Furthermore, common nutritional variables employed in daily practice (BMI, MUAC, triceps skin fold, subscapular fold and phase angle) did not show significant correlation with prognosis in our study. Therefore, PMI was the variable with the strongest association with prognostic outcomes in our study.

The pathophysiology of the loss of muscle mass and malnutrition in infants with BA is complex and of multifactorial nature (13). Apart from the aforementioned causes which involve a diminished intake due to hyporexia, altered taste, nausea, delayed gastric emptying and the subsequent early satiety, altered amino acid metabolism may cause elevated plasma tryptophan levels that increases brain serotonergic activity and subsequently downregulates hunger (31). Furthermore, fat and fat-soluble vitamins malabsorption that accompanies cholestasis may be aggravated by portal hypertension–induced enteropathy with vascular congestion and mucosal inflammation (13). Eventually, all of these mechanisms lead to a decreased intake and metabolism of nutrients, and muscle waste may ensure aggravated by the presence of GH insensitivity in these patients (14, 32).

Whereas we and other authors have shown a pivotal role of the loss of muscle mass in the prognosis of infants with BA submitted to LT (33), the challenge now is to identify sarcopenia as early as possible with validated, reliable, cost-effective and non-invasive methods. Although BIA can be an effective bedside technology for assessment of body composition in adults and children, additional research on children is needed to establish various BIA techniques as a reliable method for use in a clinical setting, especially in patients with BA and/or LT (34). Similar to BIA, ultrasonography is simple, noninvasive, and portable, and in the last few years, measuring muscle thickness in the ICU has become increasingly relevant for clinical outcomes, muscle strength and function, and nutrition status (35).

Future studies should focus on the establishment of validated cut-off points to define sarcopenia, especially in infants, on the development and validation of ultrasonography as a convenient tool, and on the research of age-appropriate muscle function tests in pediatrics (36). Furthermore, there are few studies that evaluate the possibility of muscle mass recovery in children (37) and the possible repercussions on metabolic and cognitive development that may occur. More data are needed to study the subsequent catch up in muscle mass and whether it poses a negative impact on growth, impairment of neurocognitive development and quality of life extended into adulthood.

Our study has several limitations. First, it is a retrospective study that only included patients with a CT close to surgery. This was only performed in those infants in need for a study of the vascular anatomy in preparation for LT, so there might have been a selection bias. Second, we did not have a control group of healthy infants to establish cut-off values to define sarcopenia, and reference values for psoas muscle mass of healthy children are only available for ages between 1 and 16 years (25). We solved this limitation by analyzing muscle mass as a continuous variable in our statistical workout. Third, the small sample size could have precluded us to find significant correlations of psoas muscle mass with anthropometric values or with phase angle measured by BIA. Finally, we do not have reliable data on muscle function, as the available tests commonly employed in adults have not been validated in infants.

In conclusion, the decrease in muscle mass is associated with increased morbidity in infants with BA undergoing LT, specifically with longer ICU and total hospital stays and prolonged days of mechanical ventilation. Muscle mass in these patients cannot be adequately assessed with anthropometric measurements commonly used in the clinic.

## Data Availability

The raw data supporting the conclusions of this article will be made available by the authors, without undue reservation.
